# Successful Surgical Excision of Retropharyngeal Plexiform Neurofibroma in a Non-neurofibromatosis Adult Male

**DOI:** 10.7759/cureus.43480

**Published:** 2023-08-14

**Authors:** Karina Ordaya-Gonzales, Wilfredo Ordaya, Gianpiero Gaita, Gabriel Postigo, Rodrigo W Ordaya Gonzales, Jose Arriola-Montenegro

**Affiliations:** 1 Medicine, Policlinico Centro Medico Naval, Lima, PER; 2 Otolaryngology, Centro Médico Naval Cirujano Mayor Santiago Távara, Lima, PER; 3 Surgery, Universidad Científica del Sur, Lima, PER; 4 Internal Medicine, University of Minnesota, Minneapolis, USA

**Keywords:** epiglottis, retropharyngeal space, plexiform neurofibroma, neurofibroma, otorhinolaryngology

## Abstract

Plexiform neurofibromas are benign tumors that arise from neuronal cells and are commonly associated with neurofibromatosis type 1 (NF1) patients. However, the occurrence of plexiform neurofibromas in the pharyngeal region is extremely rare. In this particular case, we report the successful diagnosis of a retropharyngeal plexiform neurofibroma in an adult male patient without a history of neurofibromatosis. The diagnosis was made using magnetic resonance imaging (MRI) and confirmed by a biopsy. Following the diagnosis, the tumor was surgically excised, resulting in a successful removal of the neurofibroma.

## Introduction

Plexiform neurofibromas (PN) are benign neoplasms that originate from neuronal cells. They consist of both neuronal and associated connective tissue, with their composition varying based on their specific location [1]. These tumors are commonly found in around 50% of individuals diagnosed with neurofibromatosis type 1 (NF1) or Von Recklinghausen's disease, but they are exceedingly rare in the general population, particularly in the pharyngeal region [2]. Due to their unique anatomical position, these tumors can lead to complications such as airway obstruction, dysphonia, odynophagia, and rhinolalia [3,4]. While they may be present since birth, plexiform neurofibromas often remain asymptomatic until adulthood, gradually becoming noticeable over time due to their slow and prolonged growth pattern [5].

In this context, we present a case report that highlights the presence of a plexiform neurofibroma in the retropharynx of a patient with no previous pathological history. This report provides insights into the clinical and radiological findings, successful surgical removal of the tumor, and histological aspects of the case.

## Case presentation

We present the case of a 51-year-old male with no significant medical history who was admitted to the hospital due to a six-month history of dysphagia, a sensation of a foreign body in the pharynx, orthopnea, rhinolalia, and significant weight loss.

**Figure 1:**
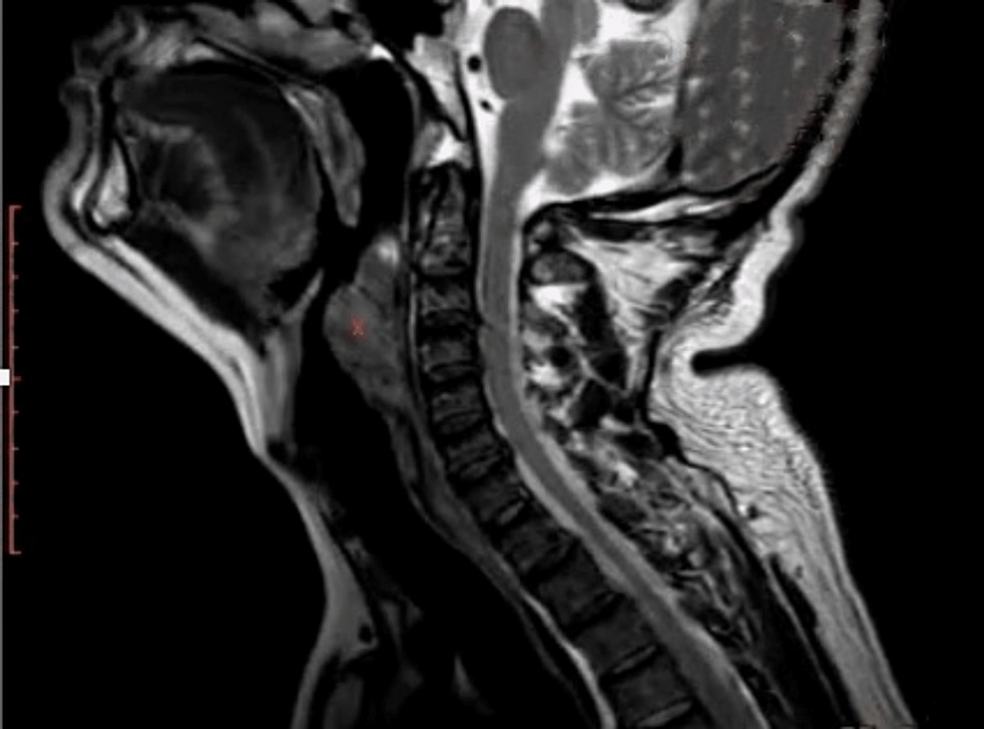
Retropharyngeal neurofibroma

During the physical examination, the vocal fold mobility was preserved and was assessed by rigid endoscopy, no swollen lymph nodes were found in the neck, and the oropharynx appeared normal. Cervical magnetic resonance imaging (MRI) was performed, revealing a tumor-like mass measuring 40 x 24 x 55 mm located in the retropharyngeal space (Figure 1).

**Figure 2:**
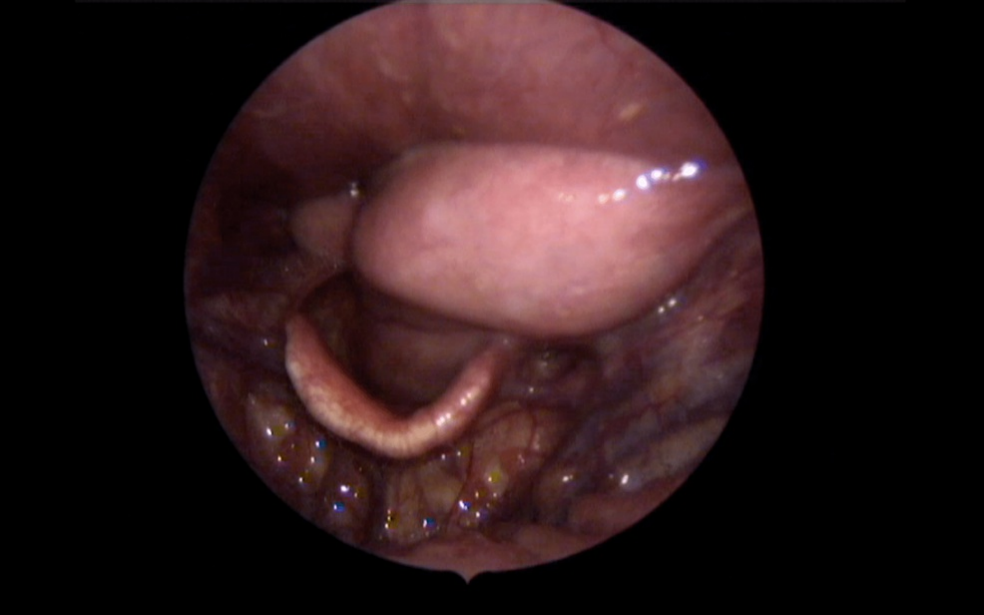
Mass in the epiglottis

Laryngoscopy confirmed that the mass was abutting the epiglottis (Figure 2). Although the mass caused partial compression of the airway, it did not invade nearby tissues. Given the complexity of the case, multiple committee meetings were held involving specialists in otorhinolaryngology, head and neck surgery, and anesthesiology. After careful consideration, it was decided to proceed with a tracheostomy due to respiratory distress, followed by indirect laryngoscopy to remove and biopsy the tumor-like mass in the retropharyngeal space.

**Figure 3:**
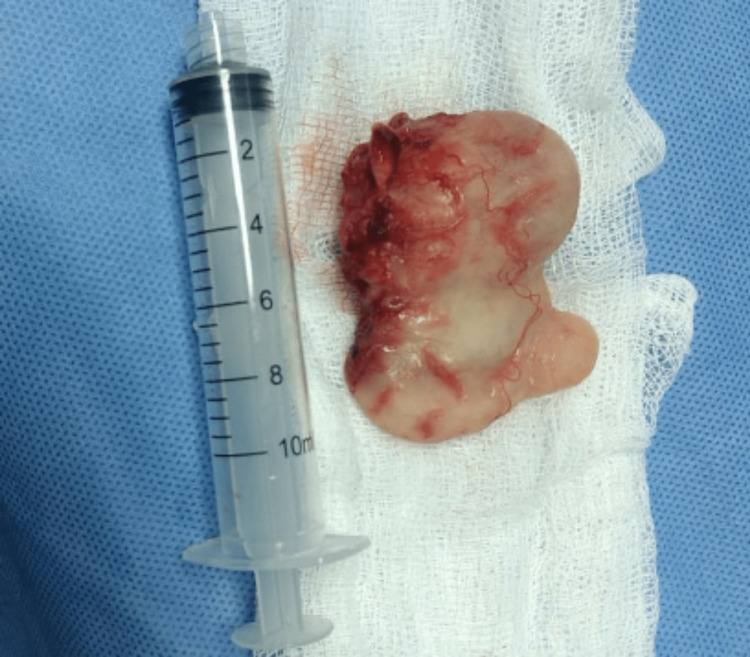
Surgical resection of retropharyngeal neurofibroma

**Figure 4:**
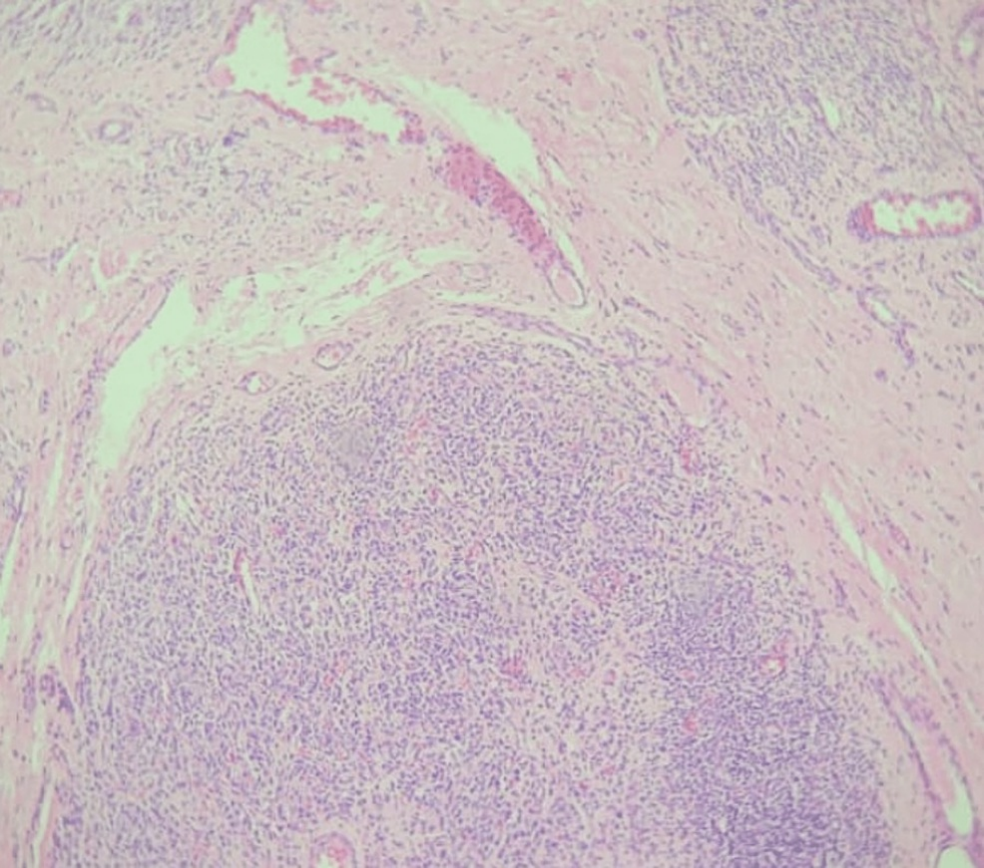
Hematoxylin and Eosin staining shows fusiform cells with packed serpentine nuclei and nerve bundles surrounded by collagenous tissue and lymphocytes

**Figure 5:**
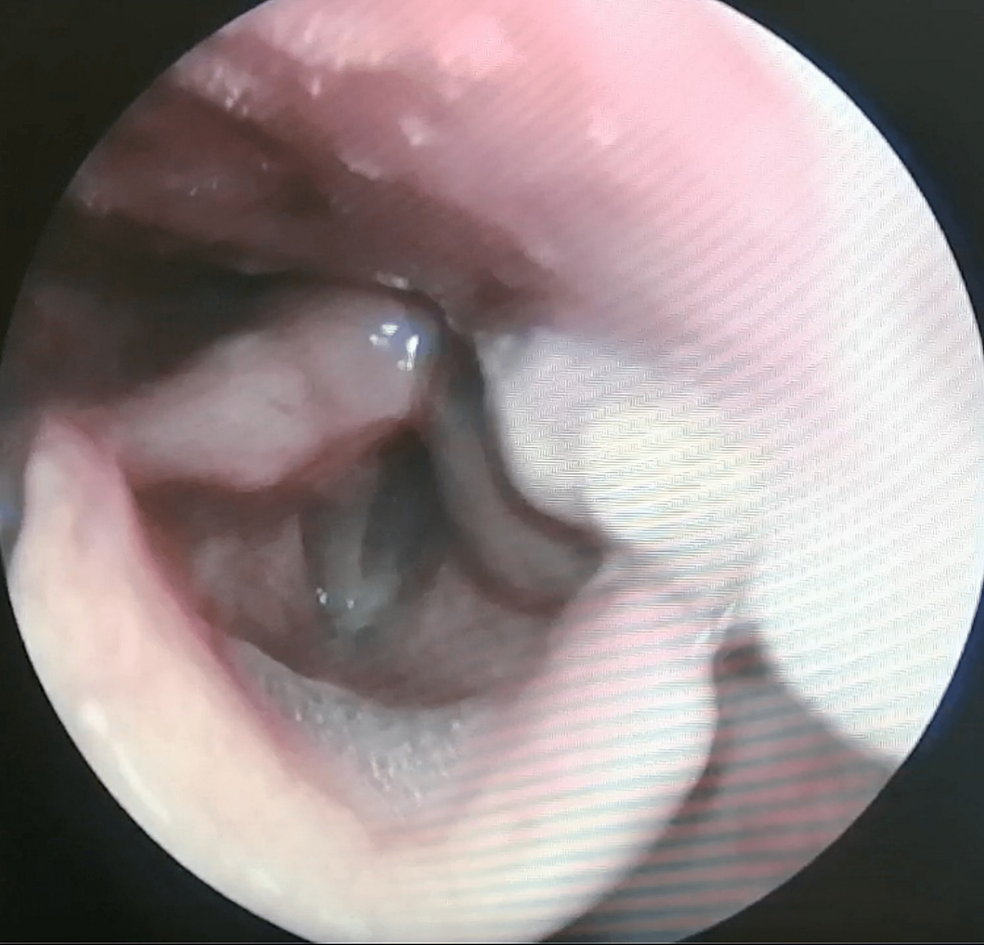
Epiglottis without neurofibroma

The surgical procedure was performed successfully without any technical complications or postoperative issues. During the postoperative course, the endotracheal tube (ETT) was successfully decannulated. The excised mass was polyp-like and measured 5 x 3 cm (Figure 3). Subsequent biopsy analysis confirmed that it was a plexiform neurofibroma (Figure 4). The patient's condition is currently stable, with resolved symptoms, and the endotracheal tube was removed after the mass excision. Follow-up laryngoscopy showed no evidence of a mass in the glottis and retropharyngeal space (Figure 5). Genetic testing for neurofibromatosis is still pending, and the patient continues to receive care and follow-up from the Otorhinolaryngology team.

## Discussion

Laryngeal neurofibroma is a rare tumor, with squamous cell carcinomas originating in the mucosa being the most common laryngeal masses that can invade surrounding tissues. Diagnosis of these tumors is typically achieved through direct examination using a rigid endoscope [6]. However, in the present case of a retropharyngeal tumor, auxiliary exams such as MRI played a crucial role in confirming the presence of the mass, determining its extent, and guiding the decision-making process regarding excision, biopsy, and surgical removal.

In individuals with a history of neurofibromatosis, tumor onset, particularly in the head and neck region, is more frequent [7]. Moreover, these tumors tend to grow slowly, presenting as masses that can involve connective tissue, mucosa, and nerve endings [8]. In our case, the neurofibroma developed within the connective tissue as an encapsulated mass. Neurofibromas are classified into three types: localized, diffuse, and plexiform, with the latter characterized by a tortuous proliferation of peripheral neurological components [9]. The clinical case at hand corresponds to a plexiform neurofibroma with a slow growth pattern.

Similarly, plexiform neurofibromas affect both sexes equally and typically manifest in patients without a history of neurofibromatosis type 1 between the ages of 30 and 60 years. Hoarseness is often the initial symptom observed, followed by dysphagia and, subsequently, dyspnea [8], mirroring the presentation in our patient. With the assistance of MRI in localizing the anatomical planes for a successful surgical intervention and the definitive diagnosis through anatomical pathology, we can conclude that our patient underwent a successful intervention for a well-diagnosed retropharyngeal plexiform neurofibroma.

## Conclusions

Plexiform neurofibromas are mostly benign neoplasms that should be considered in the differential diagnosis of head and neck tumor masses. Despite being benign, they exhibit slow growth and can develop complications, such as hoarseness, dysphagia, and dyspnea. If not diagnosed in a timely manner, these complications can lead to high mortality rates. Additionally, the assistance of auxiliary exams is important for surgical decision-making, and proper characterization of the pathological sample will help determine the origin and degree of recurrence in the patient.
